# Malaria programme personnel’s experiences, perceived barriers and facilitators to implementing malaria elimination strategy in South Africa

**DOI:** 10.1186/s12936-017-2154-8

**Published:** 2018-01-10

**Authors:** Khumbulani Welcome Hlongwana, Benn Sartorius, Joyce Tsoka-Gwegweni

**Affiliations:** School of Nursing and Public Health, College of Health Sciences, University of KwaZulu-Natal, King George Avenue, Durban, South Africa

**Keywords:** Malaria, Elimination, Eradication, Implementation, Healthcare workers, Facilitators, Barriers, South Africa

## Abstract

**Background:**

South Africa has set an ambitious goal targeting to eliminate malaria by 2018, which is consistent with the United Nations Sustainable Development Goals’ call to end the epidemic of malaria by 2030 across the globe. There are conflicting views regarding the feasibility of malaria elimination, and furthermore studies investigating malaria programme personnel’s perspectives on strategy implementation are lacking.

**Methods:**

The study was a cross-sectional survey conducted in 2014 through a face-to-face investigator-administered semi-structured questionnaire to all eligible and consenting malaria programme personnel (team leader to senior manager levels) in three malaria endemic provinces (KwaZulu-Natal, Mpumalanga, and Limpopo) of South Africa.

**Results:**

The overall response rate was 88.6% (148/167) among all eligible malaria personnel. The mean age of participants was 47 years (SD 9.7, range 27–70), and the mean work experience of 19.4 years (SD 11.1, range 0–42). The majority were male (78.4%), and 66.9% had secondary level education. Awareness of the malaria elimination policy was high (99.3%), but 89% contended that they were never consulted when the policy was formulated and few had either seen (29.9%) or read (23%) the policy, either in full or in part. Having read the policy was positively associated with professional job designations (managers, EHPs and entomologists) (p = 0.010) and tertiary level education (p = 0.042). There was a sentiment that the policy was neither sufficiently disseminated to all key healthcare workers (76.4%) nor properly adapted (68.9%) for the local operational context in the elimination strategy. Most (89.1%) participants were not optimistic about eliminating malaria by 2018, as they viewed the elimination strategy in South Africa as too theoretical with unrealistic targets. Other identified barriers included inadequate resources (53.5%) and high cross-border movements (19.8%).

**Conclusions:**

Most participants were not positive that South Africa could achieve the malaria elimination goal by 2018, citing the high cross-border movements and lack of resources as key barriers. The National and relevant Provincial Departments of Health should consider investing more time and resources in further stakeholder engagement for more effective implementation of malaria elimination strategy in South Africa.

## Background

In line with the international agenda to target malaria elimination in eligible settings, South Africa was amongst the first countries in the southern Africa to be declared as ready to pursue malaria elimination [1–3]. Some experts were very optimistic about this new goal, drawing from the success of smallpox eradication [4], while others were more pessimistic following the failure of the Global Malaria Eradication Programme (GMEP) of 1955–1969 to achieve malaria eradication. Malaria eradication is a permanent global cessation of the disease prevalence to a point, where intervention measures are no longer necessary [5, 6].

While South Africa endorsed the ‘2012–2018 Malaria Elimination Strategy’ aimed at transitioning the country to elimination phase, studies investigating malaria programme personnel’s experiences in implementing this strategy with respect to facilitators and barriers, are lacking. Such studies are needed given that malariologists are divided on the world’s readiness to pursue and achieve malaria elimination [2, 7–11]. The importance of such studies is supported by Lipsky’s theory of street-level bureaucrats. Street-level bureaucrats refers to public service workers who interact directly with the people targeted by the intervention and these workers have considerable discretion during the course of performing their jobs [12]. Without street level individuals involved in malaria control buying into these grand goals [12], the likelihood of achieving elimination is diminished, thereby increasing the risk of seeing a repeat of GMEP. The main objective of this study was therefore to investigate malaria programme personnel’s experiences with respect to the perceived barriers and facilitating factors to implementing malaria elimination strategy in South Africa.

## Methods

The study was a cross-sectional survey conducted in 2014 in all three malaria endemic provinces (KwaZulu-Natal, Mpumalanga, and Limpopo) of South Africa, through face-to-face investigator-administered semi-structured questionnaire. The questionnaire was adapted from tools used previously in other settings [13–15] and it included malaria personnel’s knowledge, perceptions and understanding of malaria elimination, their perceived roles in implementing malaria elimination, as well as perceived facilitators and barriers to malaria elimination in South Africa. Participants were also asked to identify, from a list of options, the correct WHO definition of ‘malaria elimination’. The study population was malaria programme personnel from the position of ‘team leader’ to ‘senior manager’. The continuum of positions from junior to senior positions were: spray operators–laboratory assistants–entomology assistants–Microscopists–malaria surveillance agents/case investigators–team leaders–information officers–entomologists–environmental health officers/practitioners–chief environmental health practitioners–deputy malaria programme manager–malaria programme manager–senior manager. However, some positions do not clearly follow linear pattern within and between the provinces. The inclusion of some job categories in the study was based on their level of involvement in implementing malaria elimination interventions at a strategic level. Employees at levels lower than ‘team leaders’ were excluded from this study, as their day-to-day activities were inherently operational and thus unlikely to be aware and/or involved in strategic/policy related aspects of malaria control. They are also likely to have little awareness of relevant global/regional malaria elimination strategic goals. Irrespective of job levels, all support staff, including finance, human resources, supply chain, transport officers and office administrators were also excluded, as their functions are largely administrative. In total, 167 malaria staff were eligible for inclusion, namely: 47, 79 and 41 in KwaZulu-Natal, Limpopo and Mpumalanga, respectively.

Collected data were entered into an Epi Info 7 Database, checked for errors and analysed using Stata 13.1 SE (StataCorp. 2013. Stata Statistical Software: Release 13. College Station, TX: StataCorp LP.). Association between categorical variables were assessed using the standard Pearson’s Chi square (χ2) test. If expected cell count in the cross tabulation contained fewer than 5 observations (sparse numbers) then the Fishers exact test was employed instead. A p value of < 0.05 was considered statistically significant.

## Ethical considerations

The study obtained ethical clearance from the University of KwaZulu-Natal Biomedical Research Ethics Committee (BREC) (REF: BE240/14) and approvals from the respective Provincial Departments of Health Research Committees. Subsequently, all research participants provided signed informed consent after being given detailed written and oral information about the study through participant information sheet.

## Results

### Study population characteristics

In total, 148/167 (88.6%) of eligible staff participated in the study, 40/47 (85.1%), 70/79 (88.6%), and 38/41 (92.7%) in KwaZulu-Natal, Limpopo and Mpumalanga, respectively. The research participants’ mean age was 47 years (SD 9.7, range 27–70 years) and mean years of malaria work experience was 19.4 years (SD 11.1, range < 1–42 years) (Table 1). The majority of personnel were male (78.4%), with a significantly higher percentage of males in KZN relative to other provinces (p = 0.025). Most participants had secondary level education (66.9%). Team leaders and the members of South African Malaria Elimination Committee (SAMEC) constituted 58 and 1.4% of the participants, respectively. There was a higher proportion of team leaders, Environmental Health Practitioners (EHPs) and microscopists in Limpopo compared to respondents in the other two provinces, which was reflective of the varying sizes of malaria workforce across the provinces.

**Table 1:** The overall description of the characteristics of respondents, as well as characteristics by province

Variable	Overall (n = 148)	Limpopo (n = 70)	Mpumalanga (n = 38)	KZN (n = 40)	p value
	n (%)	n (%)	n (%)	n (%)	
Age in years^a^					
≤ 30	8 (5.4)	4 (5.8)	2 (5.3)	2 (5.0)	
31–44	47 (32.0)	25 (36.2)	12 (31.6)	10 (25.0)	
45–59	76 (51.7)	31 (44.9)	21 (55.3)	24 (60.0)	0.797
≥ 60	16 (10.9)	9 (13.0)	3 (7.9)	4 (10.0)	
Home language					
Afrikaans	4 (2.7)	2 (2.9)	1 (2.6)	1 (2.5)	
English	2 (1.4)	0 (0.0)	1 (2.6)	1 (2.5)	
SePedi	22 (14.9)	20 (29.0)	2 (5.1)	0 (0.0)	
SeSotho	5 (3.4)	4 (5.8)	1 (2.6)	0 (0.0)	
SiSwati	17 (11.5)	0 (0.0)	17 (43.6)	0 (0.0)	
XiTsonga	43 (29.1)	28 (40.6)	15 (38.5)	0 (0.0)	< *0.001*
TshiVhenda	16 (10.8)	15 (21.7)	0 (0.0)	1 (2.5)	
IsiXhosa	1 (0.7)	0 (0.0)	0 (0.0)	1 (2.5)	
IsiZulu	38 (25.7)	0 (0.0)	2 (5.1)	36 (90.0)	
Gender					
Male	116 (78.4)	49 (71.0)	30 (76.9)	37 (92.5)	*0.025*
Female	32 (21.6)	20 (29.0)	9 (23.1)	3 (7.5)	
Education level attained					
Postgraduate	19 (12.8)	10 (14.5)	6 (15.4)	3 (7.5)	
Basic degree/diploma	28 (18.9)	15 (21.7)	7 (18.0)	6 (15.0)	0.413
Secondary level	99 (66.9)	44 (63.8)	26 (66.7)	29 (72.5)	
Primary level	2 (1.4)	0 (0.0)	0 (0.0)	2 (5.0)	
Job designation					
Manager	6 (4.1)	2 (2.9)	3 (7.9)	1 (2.5)	
EHP	25 (16.9)	14 (20.0)	6 (15.8)	5 (12.5)	
Entomologist	3 (2.0)	2 (2.9)	0 (0.0)	1 (2.5)	
Team Leader	87 (58.8)	42 (60.0)	23 (60.5)	22 (55.0)	
Microscopist	15 (10.1)	9 (13.0)	4 (10.5)	2 (5.0)	*0.037*
Other	12 (8.1)	1 (1.4)	2 (5.3)	9 (22.5)	
Years of experience^a^					
≤ 9	44 (29.9)	23 (33.8)	16 (41.0)	5 (12.5)	
10–19	23 (15.7)	9 (13.2)	6 (15.4)	8 (20.0)	0.237
20–29	47 (32.0)	20 (29.4)	12 (30.8)	15 (37.5)	
≥ 30	33 (22.5)	16 (23.5)	5 (12.8)	12 (30.0)	
SAMEC member^a^					
Yes	2 (1.4)	0 (0.0)	1 (2.6)	1 (2.5)	0.28
No	145 (98.6)	68 (100)	38 (97.4)	39 (97.5)	

^a^One individual with missing value i.e. N = 147 rather 148 for these variables

### Healthcare workers’ knowledge, understanding and perceptions towards malaria elimination

Participants were generally aware of the South Africa’s policy to eliminate malaria, however, less than a third (29.9%) had seen the actual copy of a policy, and only 23% had fully or partially read it. This trend was fairly consistent across the different age groups, genders and provinces (Table 2). The reading of the policy was significantly associated with job designation and level of education, whereby the reading of a policy was positively associated with senior ranking job designation (p = 0.010) and tertiary education (p = 0.042) (Table 2). Most (89%) participants contended that they were not consulted when the policy was formulated, partly because policy development is a managerial function coordinated at national level. Few (12.5%) of the consulted asserted that the policy was fully reflective of their inputs.

**Table 2:** The proportion of respondents who had fully read, partially read and not read malaria elimination policy by age, gender, work province, job designation, and education

Characteristic	Fully read it n (%)	Not read it at all n (%)	Partially read it n (%)	Total n (%)	p value
Age (n = 44)					
≤ 30	0 (0.0)	1 (20.0)	4 (80.0)	5 (100)	
31–44	2 (13.3)	3 (20.0)	10 (66.7)	15 (100)	
45–59	6 (31.6)	5 (26.3)	8 (42.1)	19 (100)	
60 +	1 (20.0)	1 (20.0)	3 (60.0)	5 (100)	0.726
Gender (n = 44)					
Female	1 (10.0)	3 (30.0)	6 (60.0)	10 (100)	
Male	8 (23.5)	7 (20.6)	19 (55.9)	34 (100)	0.602
Province (n = 44)					
KwaZulu-Natal	4 (23.5)	2 (11.8)	11 (64.7)	17 (100)	
Limpopo	1 (6.7)	6 (40.0)	8 (53.3)	15 (100)	
Mpumalanga	4 (33.3)	2 (16.7)	6 (50.0)	12 (100)	0.249
Job designation (n = 44)					
Manager	4 (66.7)	0 (0.0)	2 (33.3)	6 (100)	
EHP	1 (8.3)	2 (16.7)	9 (75.0)	12 (100)	
Entomologist	2 (100)	0 (0.0)	0 (0.0)	2 (100)	
Microscopist	0 (0.0)	1 (50.0)	1 (50.0)	2 (100)	
Team leader	1 (5.6)	7 (38.9)	10 (55.6)	18 (100)	
Other	1 (25.0)	0 (0.0)	3 (75.0)	4 (100)	*0.010*
Education (n = 44)					
Secondary	1 (5.0)	8 (40.0)	11 (55.0)	20 (100)	
Undergraduate	4 (40.0)	1 (10.0)	5 (50.0)	10 (100)	
Postgraduate	4 (28.6)	1 (7.1)	9 (64.3)	14 (100)	*0.042*

Overall, 82.5% correctly identified the ‘2018’ as target year for malaria elimination in South Africa, with a provincial break-down of 100, 87.2 and 70.8% in KwaZulu-Natal, Mpumalanga and Limpopo, respectively. Only 40.5% managed to identify the correct definition of malaria elimination. This was significantly associated with the level of education and job designation (both p ≤ 0.001), whereby correct identification was positively associated with increasing job rank and tertiary education (Table 3). The managers and EHPs, as well as participants with postgraduate education, had significantly higher chances of correctly identifying the suitable definition for malaria elimination (p ≤ 0.001). The majority of participants stated that the policy was neither sufficiently disseminated to all relevant healthcare workers (76.4%) nor properly adapted (68.9%) for the local operational context, and overall 89.1% were not confident that South Africa will successfully eliminate malaria by the year 2018.

**Table 3:** The proportion of respondents who could identify the correct WHO definition of malaria elimination from the list of seven options and this is categorised by the level of education and job designation

Possible definitions (Correct option highlighted)	Highest educational levels attained (overall n = 148)					
	Primary	Secondary	Undergraduate	Postgraduate	Total	p value
	n = 2 (%)	n = 99 (%)	n = 28 (%)	n = 19 (%)	n = 148 (%)	
Permanent global cessation of malaria…	0 (0.0)	27 (27.3)	3 (10.7)	1 (5.3)	31 (20.9)	
No local malaria transmission…	*0 (0.0)*	*26 (26.3)*	*18 (64.3)*	*16 (84.2)*	*60 (40.5)*	
Killing all malaria transmitting mosquitoes…	0 (0.0)	16 (16.2)	2 (7.1)	0 (0.0)	18 (12.2)	
Accelerated implementation of control interventions.	0 (0.0)	5 (5.1)	3 (10.7)	1 (5.3)	9 (6.1)	
All of the above	0 (0.0)	9 (9.1)	1 (3.6)	0 (0.0)	10 (6.8)	
None of the above	0 (0.0)	1 (1.0)	0 (0.0)	1 (5.3)	2 (1.4)	
Don’t know	2 (100)	15 (15.2)	1 (3.6)	0 (0.0)	18 (12.2)	*<* *0.001*

Possible definitions (correct option highlighted)	Job designation (overall n = 148)							
	Manager	EHP	Entomologist	Microscopist	Team Leader	Other	Total	p value
	n = 6 (%)	n = 25 (%)	n = 3 (%)	n = 15 (%)	n = 87 (%)	n = 12 (%)	n = 148 (%)	
Permanent global cessation of malaria…	0 (0.0)	2 (8.0)	1 (33.3)	2 (13.3)	26 (29.9)	0 (0.0)	31 (20.9)	
No local malaria transmission…	*5 (83.3)*	*21 (84.0)*	*2 (66.7)*	*3 (20.0)*	*26 (29.9)*	*3 (25.0)*	*60 (40.5)*	
Killing all malaria transmitting mosquitoes…	0 (0.0)	0 (0.0)	0 (0.0)	4 (26.7)	13 (14.9)	1 (8.3)	18 (12.2)	
Accelerated implementation of control interventions	1 (16.7)	1 (4.0)	0 (0.0)	2 (13.3)	5 (5.7)	0 (0.0)	9 (6.1)	
All of the above	0 (0.0)	0 (0.0)	0 (0.0)	1 (6.7)	8 (9.2)	1 (8.3)	10 (6.8)	
None of the above	0 (0.0)	1 (4.0)	0 (0.0)	0 (0.0)	1 (1.2)	0 (0.0)	2 (1.4)	
Don’t know	0 (0.0)	0 (0.0)	0 (0.0)	3 (20.0)	8 (9.2)	7 (58.3)	18 (12.2)	*0.000*

Options for malaria elimination definition in full, as shown in the Questionnaire: Permanent global cessation of malaria…: Permanent global cessation of malaria prevalence to a point, where intervention measures are no longer necessary

No local malaria transmission…: No local malaria transmission over a period of 3 years within a defined geographical area (Correct option—italics)

Killing all malaria transmitting mosquitoes…: Killing all malaria transmitting mosquitoes within a defined geographical area

**Fig. 1:**
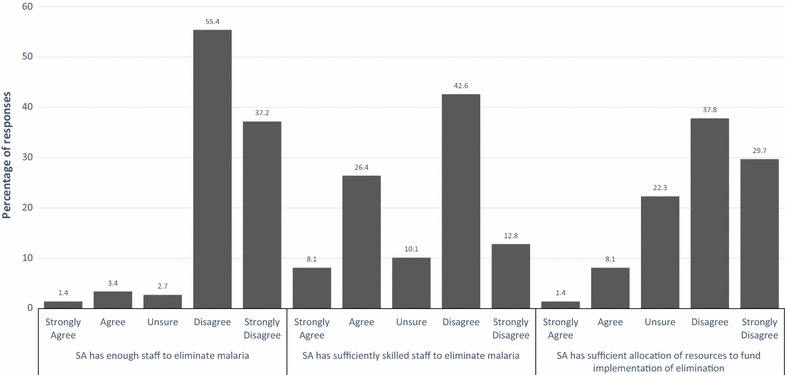
Respondents’ perceptions of adequacy of staffing to implement malaria elimination, sufficiency of skills possessed by malaria staff to implement malaria elimination and whether resources allocated to malaria programme are sufficient to fund the implementation of malaria elimination in South Africa

Most participants feared that South Africa had inadequate staff to eliminate malaria (Fig. 1). For example, when asked to rate the statement that ‘South Africa has sufficient staff to eliminate malaria’, 55.4 and 37.2% disagreed and strongly disagreed, respectively. Furthermore, a large proportion (55.4%) of participants disagreed with the statement that ‘South Africa has sufficiently skilled staff to eliminate malaria’ (Fig. 1). Participants’ views about the sufficiency of funding to implement elimination programme was more varied, with 22.3% being ‘unsure’ (Fig. 1), which could be reflective of the fact that these issues are usually discussed at treasury level. Participants’ views about the sufficiency of staff were positively associated with the level of education (p = 0.042) and malaria work experience (p = 0.016). Participants with tertiary education and less than 10 years of work experience in malaria were likely to view skilled staff as inadequate to eliminate malaria.

### Healthcare workers’ perceived roles in implementing malaria elimination strategy

**Fig. 2:**
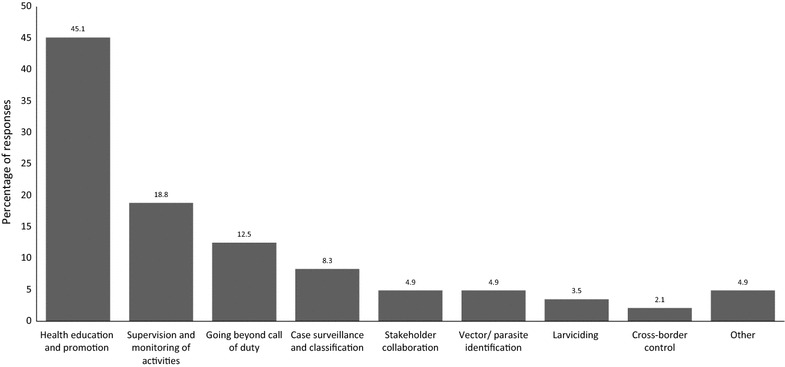
Respondents’ perceptions of their roles in implementing malaria elimination in South Africa

All but two of the participants (146/148 or 98.7%) believed that they have a role to play in implementing malaria elimination strategy, which included community health promotion/education (45.1%), supervision, monitoring and coordination of elimination activities (18.8%), and going beyond the call of duty in discharging their tasks (12.5%) (Fig. 2). However, thirty participants (20.3%) were not happy with some aspects of the malaria elimination strategy, namely: targets are too theoretical and unrealistic (63.3%), does not address resource shortage (16.7%), prioritizes surveillance over spraying (6.7%), not easy to implement (6.7%) and it does not fundamentally differ from the control strategy (6.7%). Of the 30 participants, 3 had not completed secondary education, 9 had secondary education, 8 had undergraduate qualifications, 9 had honours level qualifications and 1 had master’s degree. Their malaria work experience ranged from 3 to 40 years, with twelve being ≤ 10 years, ten were 11–20 and eight had greater than 20 years of experience. Participants’ rating of their satisfaction levels regarding their involvement in implementing malaria elimination programme in South Africa varied widely with approximately a third (31.7%) either ‘strongly dissatisfied’ or ‘somewhat dissatisfied’ with their involvement. About 45.5% of the participants were either ‘very satisfied’ or ‘somewhat satisfied’ with their involvement, and satisfaction was positively associated with senior job designation, such as EHPs, team leaders, and entomologists (p = 0.036) (Table 4).

**Table 4:** The respondents’ satisfaction levels with their involvement in the implementation of malaria elimination strategy and this rating is categorized by respondents’ age grouping, years of experience, level of education and job designation

Ratings	Age grouping (overall n = 145)					
	≤ 30	31–44	45–59	60 +	Total	p value
	n = 8 (%)	n = 46 (%)	n = 75 (%)	n = 16 (%)	n = 145 (%)	
VD	1 (12.5)	6 (13.0)	3 (4.0)	1 (6.3)	11 (7.6)	
SD	1 (12.5)	12 (26.1)	18 (24.0)	3 (18.8)	34 (23.4)	
U	1 (12.5)	9 (19.6)	20 (26.7)	4 (25.0)	34 (23.4)	
SS	4 (50.0)	16 (34.8)	23 (30.7)	6 (37.5)	49 (33.8)	
VS	1 (12.5)	3 (6.5)	11 (14.7)	2 (12.5)	17 (11.7)	
						0.769

Ratings	Years of experience (overall n = 145)					
	≤ 10	10–19	20–29	30 +	Total	p value
	n = 37 (%)	n = 24 (%)	n = 50 (%)	n = 34 (%)	n = 145 (%)	
VD	5 (13.5)	2 (8.3)	2 (4.0)	1 (2.9)	10 (6.9)	
SD	9 (24.3)	7 (29.2)	10 (20.0)	9 (26.5)	35 (24.1)	
U	8 (21.6)	4 (16.7)	14 (28.0)	8 (23.5)	34 (23.4)	
SS	12 (32.4)	9 (37.5)	16 (32.0)	12 (35.3)	49 (33.8)	
VS	3 (8.1)	2 (8.3)	8 (16.0)	4 (11.8)	17 (11.7)	
						0.897

Ratings	Highest educational levels attained (overall n = 146)					
	Primary	Secondary	Undergraduate	Postgraduate	Total	p value
	n = 2 (%)	n = 97 (%)	n = 28 (%)	n = 19 (%)	n = 146 (%)	
VD	0 (0.0)	7 (7.2)	2 (7.1)	2 (10.5)	11 (7.6)	
SD	0 (0.0)	19 (19.6)	10 (35.7)	6 (31.6)	35 (24.0)	
U	2 (100)	26 (26.8)	3 (10.7)	3 (15.8)	34 (23.3)	
SS	0 (0.0)	30 (30.9)	12 (42.9)	7 (36.8)	49 (33.6)	
VS	0 (0.0)	15 (15.5)	1 (3.6)	1 (5.3)	17 (11.6)	
						0.179

Ratings	Job designation (overall n = 146)							
	Manager	EHP	Entomologist	Microscopist	Team Leader	Other	Total	p value
	n = 6 (%)	n = 25 (%)	n = 3 (%)	n = 15 (%)	n = 85 (%)	n = 12 (%)	n = 146 (%)	
VD	1 (16.7)	3 (12.0)	0 (0.0)	0 (0.0)	5 (5.9)	2 (16.7)	11 (7.6)	
SD	3 (50.0)	9 (36.0)	1 (33.3)	6 (40.0)	15 (17.6)	1 (8.3)	35 (24.0)	
U	0 (0.0)	2 (8.0)	1 (33.3)	4 (26.7)	20 (23.5)	7 (58.3)	34 (23.3)	
SS	1 (16.7)	11 (44.0)	1 (33.3)	4 (26.7)	30 (35.3)	2 (16.7)	49 (33.6)	
VS	1 (16.7)	0 (0.0)	0 (0.0)	1 (6.7)	15 (17.6)	0 (0.0)	17 (11.6)	
								*0.036*

Ratings: *VD* very dissatisfied, *SD* somewhat dissatisfied, *U* undecided/unsure, *SS* somewhat satisfied, *VS* very satisfied

### Perceived facilitators and barriers to implementing malaria elimination strategy

Most participants positively rated their understanding of the epidemiology of malaria in South Africa. While almost half (48.3%) rated the availability of effective malaria intervention tools as ‘good’, the majority (84.4%) did not know researchers’ attitudes towards malaria elimination (Table 5). The rating of malaria research skills varied widely, so was the availability of research evidence to guide malaria elimination, the rating for political support and the cross-border collaboration with neighbouring countries (Table 5). About quarter (24.7%) and a third (34.3%) rated the availability of funds to implement elimination policy as average and poor, respectively. Nearly a third (31.3%) did not know about any research support collaboration between malaria programmes and research institutions.

**Table 5:** The respondents’ ratings of the issues likely to affect the implementation of malaria elimination in South Africa by respondents’ years of experience

Statement	Rating	Years of experience					
		< 10	10–19	20–29	30 +	Total	p value
		n = 37 (%)	n = 24 (%)	n = 51 (%)	n = 35 (%)	n = 147 (%)	
Understanding of malaria epidemiology in South Africa (overall n = 147)	Very good	8 (21.6)	7 (29.2)	9 (17.6)	11 (31.4)	35 (23.8)	
	Good	17 (45.9)	7 (29.2)	29 (56.9)	16 (45.7)	69 (46.9)	
	Average	11 (29.7)	7 (29.2)	9 (17.6)	4 (11.4)	31 (21.1)	
	Poor	1 (2.7)	1 (4.2)	3 (5.9)	2 (5.7)	7 (4.8)	
	Very poor	0 (0.0)	2 (8.3)	0 (0.0)	1 (2.9)	3 (2.0)	
	Don’t know	0 (0.0)	0 (0.0)	1 (2.0)	1 (2.9)	2 (1.4)	0.264
Availability of effective malaria intervention tools (overall n = 147)	Very good	2 (5.4)	1 (4.2)	9 (17.7)	2 (5.7)	14 (9.5)	
	Good	11 (29.7)	11 (45.8)	24 (47.1)	25 (71.4)	71 (48.3)	
	Average	19 (51.4)	10 (41.7)	13 (25.5)	5 (14.3)	47 (32.0)	
	Poor	4 (10.8)	1 (4.2)	4 (7.8)	2 (5.7)	11 (7.5)	
	Very poor	1 (2.7)	1 (4.2)	1 (2.0)	1 (2.9)	4 (2.7)	*0.018*
	Don’t Know	0 (0.0)	0 (0.0)	0 (0.0)	0 (0.0)	0 (0.0)	
Researchers’ attitudes towards malaria elimination (overall n = 147)	Very good	1 (2.7)	0 (0.0)	1 (2.0)	1 (2.9)	3 (2.0)	
	Good	0 (0.0)	1 (4.2)	4 (7.8)	3 (8.6)	8 (5.4)	
	Average	0 (0.0)	2 (8.3)	3 (5.9)	2 (5.7)	7 (4.8)	
	Poor	0 (0.0)	4 (16.7)	0 (0.0)	0 (0.0)	4 (2.7)	
	Very poor	0 (0.0)	0 (0.0)	1 (2.0)	0 (0.0)	1 (0.7)	*0.016*
	Don’t know	36 (97.3)	17 (70.8)	42 (82.4)	29 (82.9)	124 (84.4)	
Malaria research skills to conduct studies to support malaria elimination (overall n = 147)	Very good	1 (2.7)	2 (8.3)	9 (17.7)	6 (17.1)	18 (12.2)	
	Good	10 (27.0)	6 (25.0)	9 (17.7)	5 (14.3)	30 (20.4)	
	Average	10 (27.0)	4 (16.7)	11 (21.6)	3 (8.6)	28 (19.1)	
	Poor	3 (8.1)	8 (33.3)		5 (14.3)	21 (14.3)	
	Very poor	2 (5.4)	1 (4.2)	5 (9.8)	1 (2.9)	6 (4.1)	0.077
	Don’t know	11 (29.7)	3 (12.5)	2 (3.9) 15 (29.4)	15 (42.9)	44 (29.9)	
Availability of current research evidence to guide malaria elimination (overall n = 147)	Very good	1 (2.7)	1 (4.2)	7(13.7)	2 (5.7)	11 (7.5)	
	Good	7 (18.9)	3 (12.5)	12 (23.5)	7 (20.0)	29 (19.7)	
	Average	6 (16.2)	6 (25.0)	10 (19.6)	7 (20.0)	29 (19.7)	
	Poor	9 (24.3)	9 (37.5)	6 (11.8)	5 (14.3)	29 (19.7)	
	Very poor	1 (2.7)	3 (12.5)	2 (3.9)	1 (2.9)	7 (4.8)	0.159
	Don’t know	13 (35.1)	2 (8.3)	14 (27.5)	13 (37.1)	42 (28.6)	
Political leadership to support malaria elimination in South Africa (overall n = 147)	Very good	1 (2.7)	0 (0.0)	4 (7.8)	2 (5.7)	7 (4.8)	
	Good	2 (5.4)	4 (16.7)	19 (37.3)	7 (20.0)	32 (21.8)	
	Average	7 (18.9)	6 (25.0)	12 (23.5)	6 (17.1)	31 (21.1)	
	Poor	14 (37.8)	6 (25.0)	10 (19.6)	13 (37.1)	43 (29.3)	
	Very poor	8 (21.6)	5 (20.8)	4 (7.8)	2 (5.7)	19 (12.9)	*0.024*
	Don’t know	5 (13.5)	3 (12.5)	2 (3.9)	5 (14.3)	15 (10.2)	
Availability of funds to implement elimination policy (overall n = 146)	Very good	0 (0.0)	1 (4.2)	1 (2.0)	1 (2.9)	3 (2.1)	
	Good	1 (2.8)	1 (4.2)	6 (11.8)	3 (8.6)	11 (7.5)	
	Average	13 (36.1)	3 (12.5)	10 (19.6)	10 (28.6)	36 (24.7)	
	Poor	10 (27.8)	11 (45.8)	19 (37.3)	10 (28.6)	50 (34.3)	
	Very poor	4 (11.1)	5 (20.8)	8 (15.7)	5 (14.3)	22 (15.1)	0.650
	Don’t know	8 (22.2)	3 (12.5)	7 (13.7)	6 (17.1)	24 (16.4)	
Cross-border collaboration with neighbouring countries (overall n = 146)	Very good	1 (2.7)	0 (0.0)	2 (4.0)	1 (2.9)	4 (2.7)	
	Good	2 (5.4)	5 (20.8)	8 (16.0)	11 (31.4)	26 (17.8)	
	Average	7 (18.9)	4 (16.7)	15 (30.0)	4 (11.4)	30 (20.6)	
	Poor	12 (32.4)	3 (12.5)	11 (22.0)	8 (22.9)	34 (23.3)	
	Very poor	4 (10.8)	7 (29.2)	8 (16.0)	5 (14.3)	24 (16.4)	0.119
	Don’t know	11 (29.7)	5 (20.8)	6 (12.0)	6 (17.1)	28 (19.2)	
Collaborations between malaria programmes and research institutions for research support (overall n = 147)	Very good	1 (2.7)	0 (0.0)	7 (13.7)	3 (8.6)	11 (7.5)	
	Good	8 (21.6)	6 (25.0)	17 (33.3)	13 (37.1)	44 (29.9)	
	Average	10 (27.0)	6 (25.0)	6 (11.8)	6 (17.1)	28 (19.1)	
	Poor	2 (5.4)	3 (12.5)	6 (11.8)	1 (2.9)	12 (8.2)	
	Very poor	3 (8.1)	1 (4.2)	1 (2.0)	1 (2.9)	6 (4.1)	0.380
	Don’t know	13 (35.1)	8 (33.3)	14 (27.5)	11 (31.4)	46 (31.3)	
Community involvement in malaria interventions (overall n = 147)	Very Good	4 (10.8)	1 (4.2)	7 (13.7)	7 (20.0)	19 (12.9)	
	Good	17 (46.0)	8 (33.3)	22 (43.1)	13 (37.1)	60 (40.8)	
	Average	9 (24.3)	9 (37.5)	16 (31.4)	8 (22.9)	42 (28.6)	
	Poor	4 (10.8)	5 (20.8)	4 (7.8)	7 (20.0)	20 (13.6)	
	Very poor	2 (5.4)	1 (4.2)	1 (2.0)	0 (0.0)	4 (2.7)	0.618
	Don’t know	1 (2.7)	0 (0.0)	1 (2.0)	0 (0.0)	2 (1.4)	
Support from advocacy groups to maintain focus in malaria elimination (overall n = 147)	Very good	1 (2.7)	0 (0.0)	0 (0.0)	5 (14.3)	6 (4.1)	
	Good	4 (10.8)	1 (4.2)	6 (11.8)	3 (8.6)	14 (9.5)	
	Average	8 (21.6)	5 (20.8)	5 (9.8)	4 (11.4)	22 (15.0)	
	Poor	4 (10.8)	8 (33.3)	4 (7.8)	5 (14.3)	21 (14.3)	
	Very poor	5 (13.5)	3 (12.5)	1 (2.0)	1 (2.9)	10 (6.8)	*0.003*
	Don’t know	15 (40.5)	7 (29.2)	35 (68.6)	17 (48.6)	74 (50.3)	
Strategy for population movement to curb importation of malaria cases (overall n = 147)	Very good	1 (2.7)	0 (0.0)	2 (3.9)	1 (2.9)	4 (2.7)	
	Good	2 (5.4)	1 (4.2)	8 (15.7)	4 (11.4)	15 (10.2)	
	Average	6 (16.2)	2 (8.3)	3 (5.9)	7 (20.0)	18 (12.2)	
	Poor	11 (29.7)	6 (25.0)	14 (27.5)	7 (20.0)	38 (25.9)	
	Very poor	9 (24.3)	10 (41.7)	6 (11.8)	4 (11.4)	29 (19.7)	0.174
	Don’t know	8 (21.6)	5 (20.8)	18 (35.3)	12 (34.3)	43 (29.3)	

Respondents shared sentiments that the community was involved in malaria interventions and rated it as average (28.6%), good (40.8%) and very good (12.9%), respectively. About half (50.3%) of the participants did not know whether malaria programme received any support from advocacy groups to maintain focus in malaria elimination (Table 5). A substantial number of participants did not positively rate the strategy for population movement to curb the importation of malaria cases. There was a positive association between participants’ years of work experience in malaria and their ratings of availability of effective malaria intervention tools (p = 0.018), researchers’ attitudes towards malaria elimination (p = 0.016), political support (p = 0.024), and support from advocacy groups to maintain focus in malaria elimination (p = 0.003) (Table 5).

**Fig. 3:**
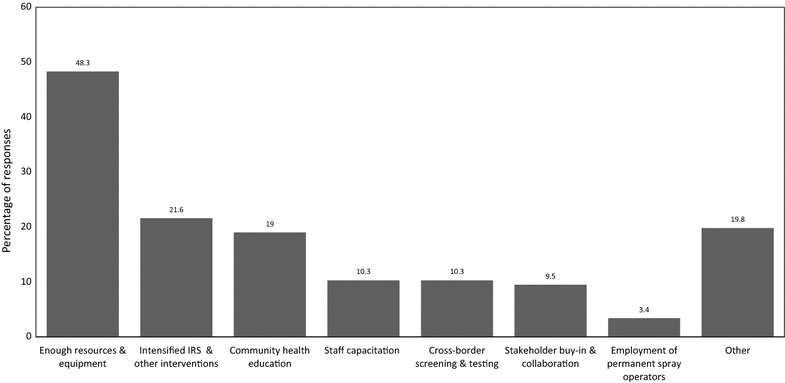
Respondents’ views on interventions they think would facilitate the successful implementation of malaria elimination in South Africa

**Fig. 4:**
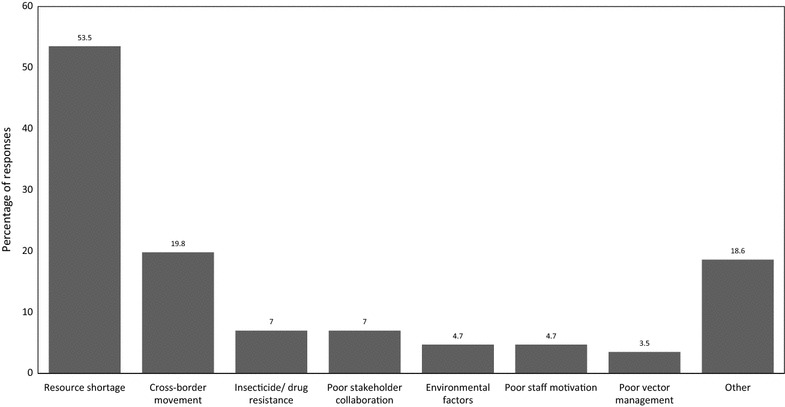
Respondents’ views of the barriers that would hinder the successful implementation of malaria elimination in South Africa

When asked to identify the interventions likely to augment the implementation of malaria elimination in South Africa, participants mentioned the following in decreasing percentages: allocation of sufficient resources and equipment (48.3%), intensified indoor residual spraying and other interventions (21.6%), community health education (19%), staff capacitation (10.3%), cross-border testing and treating (10.3%), stakeholder buy-in and collaborations (9.5%), and the employment of permanent spray operators (3.4%) (Fig. 3). The other potential enablers came to the combined total of 19.8%, which included political support, rotation of insecticides, entomological surveillance and improved procurement systems. Conversely, participants identified a range of potential barriers including resource shortages (53.5%), cross-border movements (19.8%), insecticide/drug resistance (7%), poor stakeholder collaboration (7%), environmental factors (4.7%), poor staff motivation (4.7%), and poor vector management (3.5%) (Fig. 4). The other potential barriers came to the combined total of 18.6%, which included weak procurement systems, political issues, corruption, incompetent surveillance system and poor staff capacity. Apart from definitions and targets, most participants (96.6%) held a view that malaria elimination was not different from the normal control intervention strategies implemented before the 2012 in South Africa.

## Discussion

While most malaria participants were aware about malaria elimination, few had seen or read the policy, and most had difficulty identifying the WHO definition of malaria elimination from a list of possible definitions. The majority were not convinced that the policy was developed through sufficient consultations, or was even adapted to local operational context. Most participants did not believe that the policy was disseminated to all relevant healthcare workers, neither were they optimistic that South Africa will eliminate malaria by 2018. Respondents’ perceived barriers to achieving elimination included poor cross-border collaboration, weak political support, insufficient resources and equipment, and inadequate staff capacity. However, resources and cross-border movements were seen as major priorities. Almost all participants believed that they had a role to play in implementing malaria elimination policy, including community health promotion/education, and supervision, monitoring and coordination of elimination activities. Nevertheless, some felt that the elimination strategic documents were too theoretical and unrealistic, and failed to address the resource shortage.

Contrary to most publications on malaria in South Africa, which focus on reviews or use secondary/routine data from malaria programmes [and not primarily designed for research purposes] [9, 16–25], this study provides malaria programme personnel’s perspectives of malaria elimination strategy in South Africa using primary data. Chilundo et al. [26] have asserted that analysis of routine malaria data raises serious data quality issues and lack perspectives. Another important strength of this study was that is encompassed all the provinces (and hence personnel) in South Africa affected by malaria. While this study has provided a broad overview of the issues affecting the implementation of malaria elimination strategy in South Africa from the perspectives of malaria programme personnel as frontline healthcare workers, the perspectives of the affected communities are missing. This is an important limitation given the impact community involvement and support has on the effectiveness of the interventions as observed in the successful implementation of malaria elimination strategy in Vanuatu Islands [27]. A study conducted by Govere et al. [28] in Mpumalanga Province, South Africa, found that the community members usually become reluctant to accept malaria interventions once malaria is no longer perceived to be a problem.

Early in the global malaria elimination debate South Africa was identified as ready to target malaria elimination [9, 23]. Therefore, it was not surprising that malaria programme personnel participating in this study were generally aware about the South Africa’s intention to eliminate malaria. However, 17.5% could not correctly recognize the country’s target year for elimination. Furthermore, less than a third of the study participants had seen a copy of the policy, and of these only 20.5% had fully read the document. That was concerning, since one would argue that the policy should be a reference document for healthcare workers to monitor their interventions and progress against the policy objectives. The assertion by most participants that they were never consulted when the policy was formulated was also concerning and inconsistent with modern governance practices [29]. It was unsurprising that only 40.5% of the participants managed to identify the correct definition of malaria elimination, given the fact that the majority had neither seen nor read the policy. Most research participants felt that the policy had not been sufficiently disseminated to all relevant healthcare workers, and none of the participants, irrespective of the occupational seniority level, were in possession of the endorsed final elimination policy document. Understandably, the elimination document should be constantly evolving to incorporate real-time events. Apart from the strategy document that was approved at Ministerial level, there is no endorsed final document.

While substantial reduction in malaria transmission in South Africa led to the country being earmarked as ready for elimination [1–3], evidence has shown that countries achieving these reductions can be the victim for their own successes through reduced budgets allocated for malaria, and decreased index of malaria suspicion, making it difficult for healthcare workers to detect parasites and administer treatment [30–34]. This can be further complicated by community’s complacency towards the uptake of malaria interventions, as they perceive it not to be a problem [28, 35]. Research has revealed that averting the last sporadic malaria cases and deaths would require a considerable increase in funding, thus making it a less efficient way of using limited health resources in the context of competing health priorities, such as HIV/AIDS [36]. Furthermore, Zelman et al. [37] found that South Africa experienced a substantial reduction in malaria financing from both external and domestic sources, between 2005 and 2010. However, external funding in South Africa has mainly been for operational research, which was still considered helpful in addressing research questions faced by malaria programmes in their day-to-day operations. Participants in this study were also equally concerned about malaria budget allocation, a phenomenon consistent in other countries attempting elimination [4, 31, 38]. For example the diversion of funds from malaria to other perceived health priorities in Sri Lanka prevented the country from eliminating malaria in the early 1960s, thus taking several more decades before interruption of local transmission occurred once more [37].

While eliminating malaria in South Africa is not practically impossible, studies investigating malaria programme personnel’s conceptualization of elimination, including their perceived roles, as well as facilitators and barriers to policy/strategy implementation are fundamental. Such studies should complement the proposition by Moonen et al. [39] that elimination should be preceded by a thorough quantitative feasibility assessment of a country’s readiness to eliminate malaria, which includes: malaria epidemiology, resource availability, public health system and the status of malaria control in neighbouring areas/countries. South Africa conducted a thorough malaria programme review in 2009 just prior to moving to an elimination agenda [40]. However, this study, which was conducted 5 years later, raised some similar issues, including the shortage of skilled human capacity. The importance of studying healthcare workers is supported by Lipsky’s theory of street-level bureaucrats [12], which argues that frontline public workers make decisions based on professional discretion, available resources, costs and practical arrangements, and such decisions modify how the policy is implemented. Therefore, it is important to ensure that malaria programme personnel in South Africa sufficiently appreciate the context and value of malaria elimination goal in order to push for proper implementation.

Findings on barriers to implementing malaria elimination strategy in this study are comparable to user-fee exemption policy in health facilities in Ghana [12], health providers’ implementation of abortion policy in Ghana [41] and the barriers to scaling up health interventions in low and middle income countries [42]. Resource constraints, attitudes towards the policy, lack of engagement of local implementers, lack of technical consensus, poor leadership, and health systems capacity, emerged as key reasons for poor implementation and were common across these studies [12, 41, 42]. Congruent with the results of this study, Woyessa et al. [43] identified the shortage and poor distribution of well-trained and adequately-motivated healthcare workers in the malaria programme as part of the challenges facing elimination efforts in Ethiopia. Participants in this study were not optimistic that South Africa would successfully eliminate malaria in 2018, and this was supported by projections by Silal et al. [44], which suggested that the target would not be achieved by the target year. The upsurge of malaria cases and malaria deaths from 6385 cases and 58 deaths in 2015/2016 malaria season to 9478 cases and 76 deaths by March 2017 attests to the difficulties the country faces in achieving the now close 2018 target [45]. It is worth noting that KwaZulu-Natal has been in elimination for the past decade and appears to be on track to achieve sub-national malaria certification, hence the respondents from this province were expected to be more positive about the potential to eliminate malaria in their responses. However, after data collection for this study had been completed, the country has embarked on a foci-clearing programme and all levels of the malaria management hierarchy received training and information on the malaria elimination strategy and the need for continued and improved surveillance.

## Conclusions

This study revealed multiple potential factors affecting the implementation of malaria elimination in South Africa, and provides empirical evidence the Department of Health can use to strengthen the implementation of the strategy. Most participants already considered the country’s goal to eliminate malaria by 2018 as unachievable and this has been exacerbated by the recent upsurge of malaria in South Africa. Lastly, these results provide uniquely important insights into the issues affecting the implementation of malaria elimination in South Africa from the perspectives of malaria programme personnel. The issues raised in this study may be useful for countries with similar settings, targeting to eliminate malaria, especially since malaria programme personnel’s perspectives in the implementation of malaria control/elimination strategy have not previously been viewed as an attractive researchable phenomena.

## Abbreviations

BREC: Biomedical Research Ethics Committee
GMEP: Global Malaria Eradication Campaign
KZN: KwaZulu-Natal
MDGs: Millennium Development Goals
SAMEC: South African Malaria Elimination Committee
WHO: World Health Organization
